# The diagnostic test accuracy of rectal examination for prostate cancer diagnosis in symptomatic patients: a systematic review

**DOI:** 10.1186/s12875-018-0765-y

**Published:** 2018-06-02

**Authors:** Daniel Jones, Charlotte Friend, Andreas Dreher, Victoria Allgar, Una Macleod

**Affiliations:** 1 Hull York Medical School, Hertford Building, University of Hull, Cottingham Road, Hull, HU6 7RX UK; 2 Goethe University Frankfurt, Theodor-W.-Adorno-Platz 1, 60323 Frankfurt am Main, Germany; 3 Faculty of Health Sciences, University of York, Heslington, York, YO10 5DD UK

**Keywords:** General practice, Digital rectal examination, Prostate Cancer, Primary care, Early diagnosis

## Abstract

**Background:**

Prostate cancer is the most common cancer in men in the UK. NICE guidelines on recognition and referral of suspected cancer, recommend performing digital rectal examination (DRE) on patients with urinary symptoms and urgently referring if the prostate feels malignant. However, this is based on the results of one case control study, so it is not known if DRE performed in primary care is an accurate method of detecting prostate cancer.

**Methods:**

The aim of this review is to ascertain the sensitivity, specificity, positive and negative predictive value of DRE for the detection of prostate cancer in symptomatic patients in primary care.

CENTRAL, MEDLINE, EMBASE and CINAHL databases were searched in august 2015 for studies in which a DRE was performed in primary care on symptomatic patients and compared against a reference diagnostic procedure.

**Results:**

Four studies were included with a total of 3225 patients. The sensitivity and specificity for DRE as a predictor of prostate cancer in symptomatic patients was 28.6 and 90.7%, respectively. The positive and negative predictive values were 42.3 and 84.2%, respectively.

**Conclusion:**

This review found that DRE performed in general practice is accurate, and supports the UK NICE guidelines that patients with a malignant prostate on examination are referred urgently for suspected prostate cancer. Abnormal DRE carried a 42.3% chance of malignancy, above the 3% risk threshold which NICE guidance suggests warrants an urgent referral. However this review questions the benefit of performing a DRE in primary care in the first instance, suggesting that a patient’s risk of prostate cancer based on symptoms alone would warrant urgent referral even if the DRE feels normal.

## Background

Prostate cancer is the most common cancer amongst men with 41,736 cases diagnosed in the UK in 2011. Over the last 35 years, the incidence of prostate cancer has more than tripled, though much of this increase is likely to be due to the increasing use of prostate specific antigen (PSA) blood tests. The mortality rate from prostate cancer in the UK is falling after reaching a peak in the 1990s, but in 2012, over 10,000 men died of prostate cancer. Survival from prostate cancer is relatively good with a five-year survival rate of 85% [1].

There has been considerable debate about the benefits and harms of early diagnosis of prostate cancer, with much of the discussion focused around the use of PSA. Evidence suggests survival is closely related to stage at diagnosis, with 100% five year survival in patients diagnosed with the earliest stage disease compared to less than 33% five year survival if diagnosed at the latest stage [1]. This suggests that early diagnosis of prostate cancer is important. Certainly once a patient is symptomatic, there seems to be little benefit in delaying the diagnosis.

Asymptomatic screening using PSA is undertaken, and accepted in some countries, including the US, however the U.S. Preventative Services Task Force recommend a discussion on the potential benefits and harms of PSA screening, stating that screening offers a “small potential benefit of reducing the chance of dying of prostate cancer” but also highlighting that “many men will experience potential harms of screening” [2]. In the UK, screening is not recommended routinely, instead Public Health England runs a ‘prostate cancer risk management program’ in which patients who are concerned about prostate cancer are able to have a PSA test after a discussion with a GP on the benefits and harms of the test in order to make an informed choice [3]. As a result most prostate cancers in the UK are identified when patients present to general practice with a symptom suspicious of prostate cancer such as nocturia or urinary frequency. It is also worth noting however that there is a diagnostic challenge as both urinary tract infections and benign prostatic hypertrophy often present in similar ways and are much more common diagnoses [4, 5].

The National Institute for Health and Care Excellence (NICE) has recently updated the guidance on recognition and referral of suspected cancer in the UK [6]. The latest NICE guidelines give recommendations on the recognition and referral of prostate cancer. These guidelines state patients should be referred urgently if the prostate feels malignant on digital rectal examination and recommends performing digital rectal examination (DRE) on patients presenting with any lower urinary tract symptoms including nocturia, urinary frequency, hesitancy, urgency or retention [6]. A recommendation supported by Walsh et al. who reviewed DREs undertaken in primary care and urology clinics for the diagnosis of prostate cancer and concluded that DRE is a key part of the assessment [7]. However, the evidence base for DRE in symptomatic patients is poor, with NICE guidelines documenting only one case control study by Hamilton et al. [4]. As a result it is not known if DRE performed in primary care is an accurate method of detecting prostate cancer, or what the risk of prostate cancer is, if the general practitioner deems the prostate to be malignant on examination.

## Methods

The aim of this review was to evaluate the effectiveness of DRE performed by general practitioners as a predictor of prostate cancer in symptomatic patients in a primary care setting according to the latest NICE guideline recommendations.

The reporting of this systematic review follows the recommendations of the PRISMA (Preferred Reporting Items for Systematic Reviews and Meta-Analyses) statement [8]. A protocol was developed and registered in the PROSPERO register of systematic reviews (registration number PROSPERO 2015:CRD42015025764) [9].

### Search strategy

A search was undertaken for empirical research using MEDLINE, CINAHL, EMBASE and the Cochrane Register of Controlled Trials (CENTRAL). Each database was searched from commencement to August 2015. Grey literature was searched using the OpenGrey database. Citations of all potentially relevant reviews and research papers were hand searched. No date or language restrictions were applied to the database searches.

The following terms were used in the search all databases: prostate cancer, DRE, primary care. The search strategy and protocol and be accessed here http://www.crd.york.ac.uk/PROSPERO/display_record.asp?ID=CRD42015025764. Two reviewers (CF and AD) independently screened the title and abstract of all articles identified by the search to determine eligibility. Full texts were obtained for all potentially relevant articles and these were independently assessed by two authors (CF and AD) to determine eligibility. Final inclusion was determined by agreement between both reviewers. If no consensus was reached, a third study author (DJ) was consulted.

### Eligibility criteria

We searched for studies that evaluated the use of DRE in primary care for the detection of prostate cancer. To be included in the review studies had to meet three inclusion criteria: studies should be randomized controlled trials, case control or cohort studies; they needed to include symptomatic patients with any of the symptoms listed in the NICE guidelines for referral of prostate cancer; and they studies needed to be conducted in primary care setting. Studies undertaken in secondary care or screening studies of asymptomatic patients were excluded. As this was a review of a diagnostic procedure the studies should compare DRE to a reference test. The reference for this review was histological diagnosis of prostate cancer. The NICE guidelines do not define an abnormal DRE so definitions from all included studies were considered.

### Outcomes

The primary outcomes were the sensitivity, specificity, positive (PPV) and negative predictive value (NPV) of DRE in primary care for the detection of prostate cancer in symptomatic patients. This was calculated using the Meta-DiSc software [10]. Secondary outcomes were cost-effectiveness and adverse effects as a result of the intervention. Heterogeneity is almost always presumed in diagnostic test accuracy systematic reviews, and hence, a random-effects model was used [11].

### Data extraction

Two reviewers (CF and AD) independently extracted data from included articles using a pre-defined data extraction form. Disagreements were resolved by discussion between the two reviewers with a third reviewer (DJ) consulted if necessary. Data were extracted on study participants including age, ethnicity, setting and symptoms, the studies inclusion and exclusion criteria, the information on the DRE and gold standard test of the study and study outcomes.

### Quality assessment

The Newcastle Ottawa quality assessment scale [12] was used to evaluate the quality of included studies. This scale is recommended by the Cochrane Handbook for assessing methodological quality of non-randomised studies and was chosen as it was highlighted as a simple tool to apply using eight assessment domains [13].

Hamilton [4] was a case control study and was determined to be of high methodological quality. The study adequately defined a case, stating they were identified from the cancer registry at the Royal Devon and Exeter Hospital and controls were selected from the community with no history of disease. The study adequately controlled for age and location. Exposure for both cases and controls was ascertained from the GP and hospital records. All cases and controls were included in the final results with no drop outs.

Three cohort studies [14–16] were included and were judged to be of high methodological quality. All were representative of the average age of men with prostate cancer in the community. Ascertainment of exposure for all studies was determined from a secure patient medical record. Participants with a history of prostate cancer were excluded in all studies. All studies controlled for symptomatic patients, location and performance of DRE. However, it should be noted that none of the cohort studies included documentation of how biopsy was taken and whether the individual performing the biopsy was blinded of symptoms and DRE findings.

## Results

### Study characteristics

**Fig. 1:**
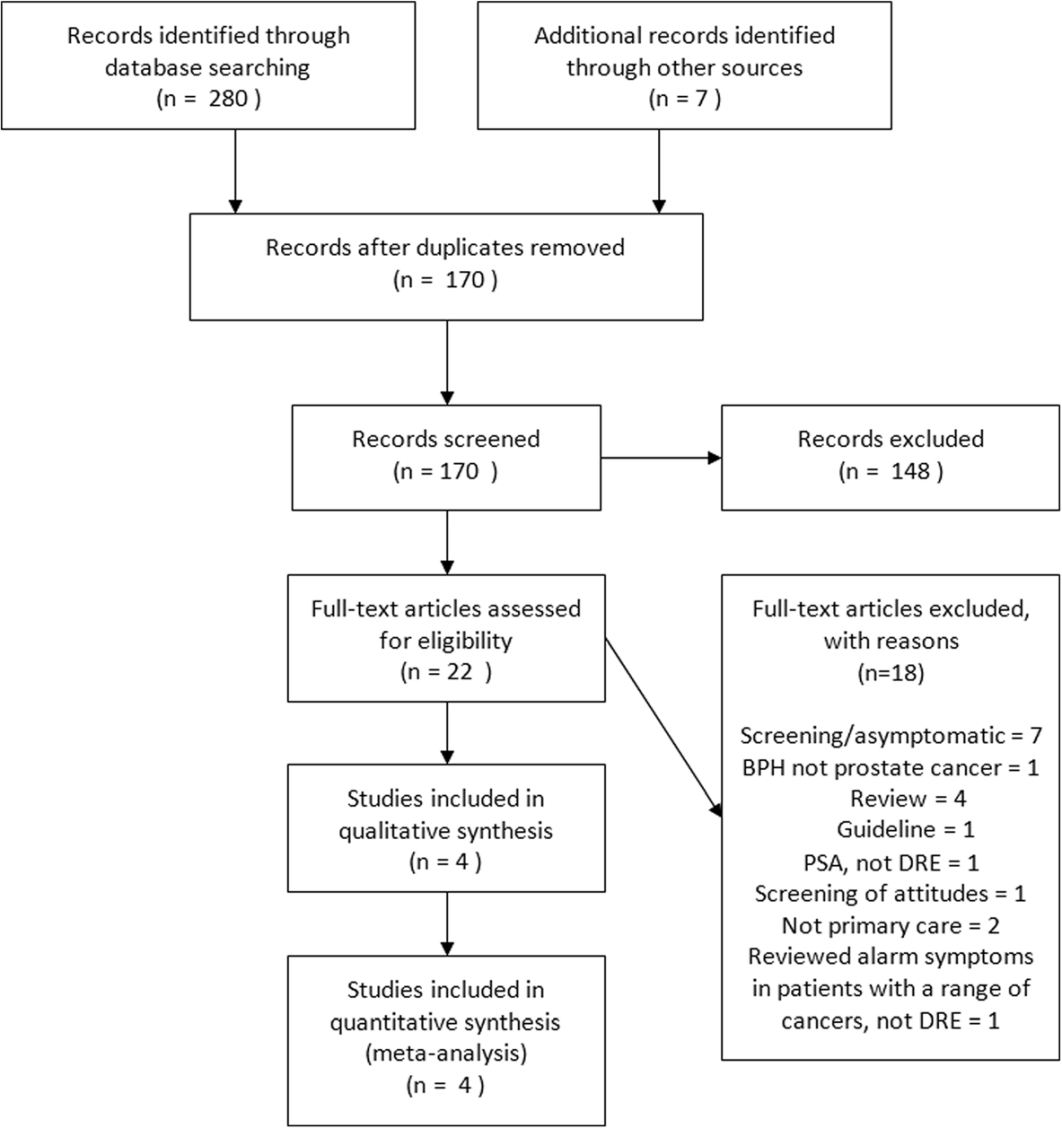
PRISMA Diagram

Four studies met the inclusion criteria and were included in the review (see Fig. 1). The characteristics of the included studies are shown in Table 1. Three of the studies were cohort studies [14–16] and one was a case control study [4]. Two of the studies were conducted in the US [14, 15], one was conducted in the UK [4] and one in Spain [16]. A total of 3225 patients were included in the four papers. The largest study had 1297 participants [4] and the smallest had just 82 [16]. The age of participants in included studies ranged from 40 to 89. All studies documented the symptoms suffered by patients with the exception of Issa [14] who used the international prostate symptom score (IPSS). All four papers included patients with at least one of symptoms listed in the NICE cancer guidelines, however these were different in each paper. Three of the studies explicitly defined an ‘abnormal DRE’ with the exception of Gelabert Mas [16]. The three papers used very similar definitions of an abnormal prostate. Hamilton et al. state that “hard, craggy or nodular glands were classified as malignant” [4], Issa et al. state that “DRE findings were classified as abnormal in the presence of prostate induration and/or nodularity” [14] and Mettlin et al. state that “a suspicious DRE outcome is defined by the presence of significant induration, nodularity or asymmetry” [15].

**Table 1:** Characteristics of included studies

Study ID	Country	Evidence level	Methods	Number of participants	Age range (years)	Index test	Reference	Outcomes
Mettlin 1991	USA	2b	Prospective cohort study	1218	55–70	PSA, DRE and TRUS	Biopsy	Sensitivity, specificity and PPV of DRE, PSA and TRUS.
Gelabert Mas 1997	Spain	2b	Prospective cohort study	82	> 50	PSA/DRE	Biopsy	Sensitivity, specificity, and PPV of DRE and PSA.
Hamilton 2006	UK	4	Case control	1297	≥ 40	PSA/DRE	Diagnosis of prostate cancer	PPV of symptoms, DRE and PSA.
Issa 2006	USA	2b	Retrospective cohort study	628	40–89	PSA/DRE	Biopsy	Sensitivity, specificity, PPV and NPV of DRE and PSA.

### Diagnostic accuracy of DRE

**Fig. 2:**
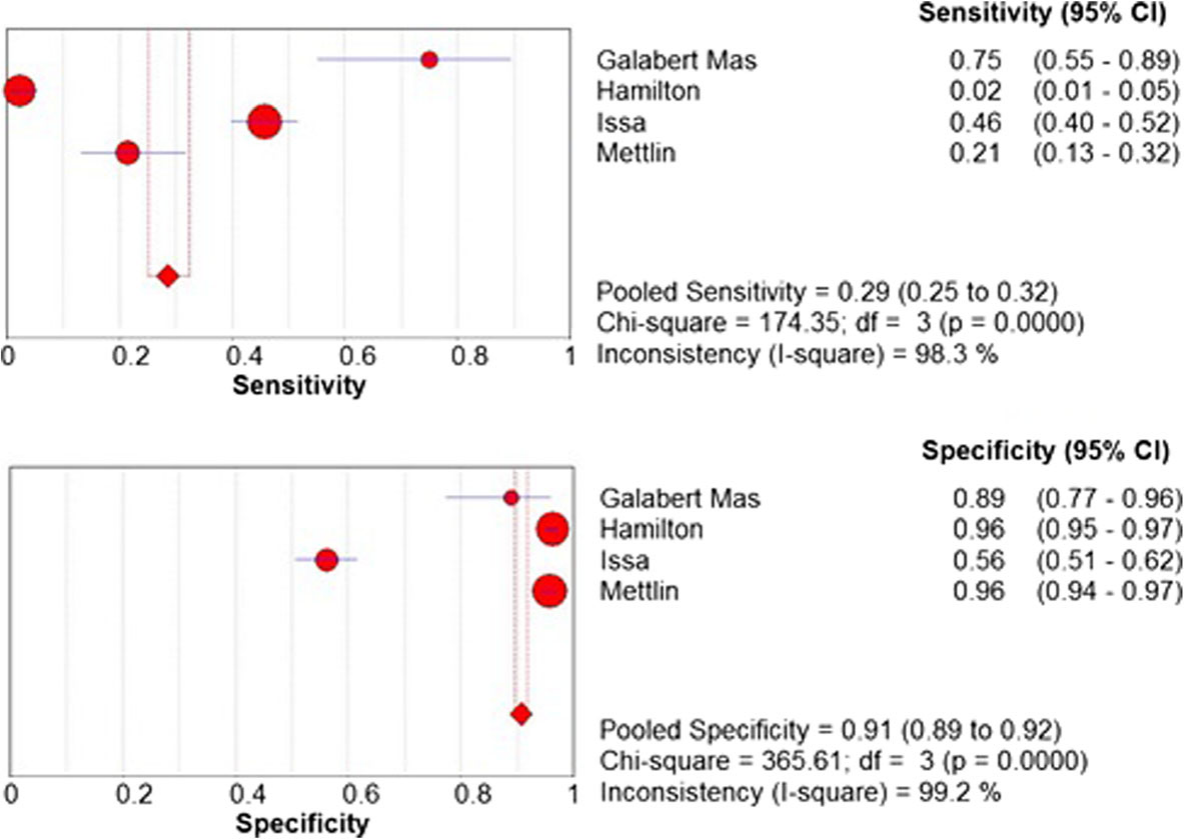
Pooled sensitivity and specificity for DRE as a predictor of prostate cancer in symptomatic patients

There were 3225 participants included from 4 different studies. For each of the included studies we were able to calculate a 2 × 2 table for reference test (biopsy / diagnosis of prostate cancer) results versus the diagnostic test (DRE). This data was then combined to give an overall sensitivity, specificity, PPV and NPV. This was calculated using Meta-Disc software. Overall, the pooled sensitivity and specificity for DRE as a predictor of prostate cancer in symptomatic patients was found to be 28.6% (95% CI 25.1–32.3%) and 90.7% (95% CI 89.5–91.8%), respectively. These results are shown in Fig. 2. The pooled PPV and NPV were found to be 42.3 and 84.2%, respectively. There was no relevant data extractable regarding secondary outcomes of adverse events or cost effectiveness.

As three out of the four studies were cohort studies, we performed a sub-analysis of the cohort studies alone. This produced a sensitivity of 42.7% (95% CI 37.8–47.7%), a specificity of 86.7% (95% CI 84.9–88.4%), a PPV of 46.1% and a NPV of 85.1%. Showing that excluding the case control study did not significantly affect the results.

## Discussion

To the authors´ knowledge, this is the first review to evaluate the specificity, sensitivity, positive and negative predictive value of DRE as a predictor of prostate cancer in symptomatic patients in a primary care setting.

With the release of the new cancer referral guidelines in the UK the threshold of risk for referral for possible cancer was reduced from 5 to 3%. This suggests that all patients with signs and symptoms which carry a risk of cancer greater than 3% should be referred urgently to secondary care. This review found the pooled positive predictive value of DRE to detect prostate cancer was 42.3%. This suggests that if a patient with symptoms suggestive of prostate cancer presents to primary care and has an abnormal feeling prostate examination, then that patients risk of cancer is 42.3%. This clearly warrants an urgent referral for suspected cancer and supports the UK NICE guidelines.

However, the pooled sensitivity in this review was low, 28.6% suggesting that many patients diagnosed with prostate cancer do not have an abnormal DRE. In addition to the low sensitivity, the pooled negative predictive value was 84.2%, this suggests that symptomatic patients who present to primary care and have a normal prostate examination still have a risk of cancer of 15.8%, which above the 3% risk threshold suggested by NICE. These findings show that patients in these included studies should be urgently referred for suspected prostate cancer regardless of the DRE result. This suggests that DRE performed in primary care is an unnecessary investigation, adding little to the decision to refer, which should be made on the basis of symptoms alone. In addition to this, it is possible that DRE may delay the patient in seeking help with some qualitative research finding that the prospect of a DRE may deter some men from seeking medical help for symptoms suggestive of prostate cancer and prostate cancer screening [17–19]. This qualitative research suggests that performing DRE in primary may in fact be delaying the diagnosis of prostate cancer.

The UK NICE guidelines make their recommendations to refer patients with a prostate deemed malignant on examination on the evidence from just one study. This systematic review includes four studies which consider the effectiveness of DRE for diagnosing prostate cancer in symptomatic patients in primary care and thus provides stronger evidence for performing DRE in these patients. However, the majority of the available literature concentrates on using DRE as a screening test in unselected asymptomatic patients. More research on the effectiveness of DRE in primary care would help to provide further evidence for the NICE guidelines and ensure that DRE is a useful investigation when performed by GPs.

Whilst the included studies were judged to be of high methodological quality it is difficult to draw conclusions based on a small number of studies, all of which were published over ten years ago, are of low impact and are largely heterogeneous. In addition, there are limitations of including case control studies in reviews of diagnostic test accuracy as they tend to overestimate diagnostic accuracy. In addition to this Hamilton et al. included patients diagnosed with prostate cancer by a urologist without having a biopsy which means not all participants in the study received the same index test. Whilst this clinically makes sense and is common in studies in which the reference test is invasive, it is nonetheless a limitation of the study. These limitations were investigated using a sensitivity analysis which excluded the Hamilton et al. study, it was found that excluding the case control study did not significantly affect the results.

To be included in this review the papers had to include patients with at least one of the symptoms suggested by NICE. However, in each paper the combination of symptoms in the patients was different and in some was poorly defined.

Due to the nature of using secondary data, there were some papers in which full information was not available. It was agreed in each study the DRE had been performed in primary care, however it was often not clear who had performed the examination. In addition to this, only three of the four studies adequately defined an abnormal DRE, the forth study did not provide a definition of abnormal or positive DRE.

One of the papers (Issa 2006) caused some difficulty in analysis due to a possible typing error. In the text of the study they state symptoms were classified as mild moderate or severe using IPSS with mild symptoms classified between 1 and 7, however in the results they present mild symptoms as 0–7, this could mean patients with an IPSS score of 0, who may be asymptomatic were included in the results. Clarification was sought from the authors however no response was received.

The vast majority of existing literature focuses on the use of DRE as a screening tool. A Cochrane review on screening for prostate cancer included five studies and found that screening did not significantly decrease prostate cancer-specific mortality [20]. This review supports the recommendations of NICE which suggests that GPs refer patients urgently on the basis of an abnormal DRE. The NICE recommendation was made on the basis of the Hamilton 2006 paper which is included in this review. There is also qualitative literature to suggest that the prospect of a DRE may deter men from presenting to primary care.

## Conclusion

The most recent UK NICE guidelines recommend that general practitioners undertake a digital rectal examination on all patients with lower urinary tract symptoms. The guidelines suggest referring patients under the two week wait referral pathway if the prostate feels malignant. This is the first review to look at the accuracy of digital rectal examination by general practitioners for the diagnosis of prostate cancer in symptomatic patients. We found only four studies on the effectiveness of DRE for the diagnosis of prostate cancer in symptomatic patients in primary care, with much of the available literature focusing on screening. DRE is widely used and recommended for the assessment of patients in primary care. It is a simple, safe and cost-effective diagnostic tool, The findings of the review support the NICE guidelines and recommend urgent referral for suspected cancer in patients with an abnormal DRE, however the review casts doubt on the use of DRE as a diagnostic tool in primary care due to the low sensitivity and negative predictive value. More research is needed to assess its effectiveness in diagnosing prostate cancer in symptomatic patients.

## Abbreviations

DRE: Digital rectal examination
NICE: National Institute for Health and Care Excellence
PRISMA: Preferred Reporting Items for Systematic Reviews and Meta-Analyses
PSA: Prostate specific antigen
